# Reciprocal adaptation is critical in enhancing *S. aureus* and *P. aeruginosa* biofilm biomass

**DOI:** 10.1007/s00203-026-05020-3

**Published:** 2026-06-22

**Authors:** Xiaohan Sun, Clare M. Cooksley, Muhammed Awad, Emma F. Barry, Alkis J. Psaltis, Peter-John Wormald, Sarah Vreugde

**Affiliations:** 1 Department of Surgery-Otolaryngology, Head and Neck Surgery, Central Adelaide Local Health Network, Woodville, South Australia Australia; 2 Adelaide Medical School, Faculty of Health and Medical Sciences, The University of Adelaide, Adelaide, South Australia Australia

**Keywords:** Antibiotic resistance, Biofilms, Chronic rhinosinusitis, *Pseudomonas aeruginosa*, *Staphylococcus aureus*

## Abstract

Polymicrobial communities impose a great challenge for clinical management of chronic infections. It is a consensus now that microbes exist as aggregated colonies shielded within polymeric matrix. Within this matrix more than one bacterial species can exist either in symbiotic or rival relationships. Herein, we investigated the host-specific interspecies interactions between *Staphylococcus aureus* and *Pseudomonas aeruginosa* in chronic rhinosinusitis (CRS). The indirect interaction between the two species was assessed using Transwell co-culture chambers, where *S. aureus* and *P. aeruginosa* (*n* = 3 each) derived from CRS patients were cultured in separate chambers that allowed exchange of soluble factors. Later the biofilm biomass of each species was evaluated and compared to single species biofilm. Further, the influence of the co-culture conditions on antibiotic tolerance was evaluated. When derived from the same patient, co-cultured bacteria increased the biofilm biomass of each other significantly by 3.0-4.9 fold (*p* < 0.01) and exhibited higher tolerance to amikacin compared to co-cultures of isolates from two different patients and monocultured biofilms. Moreover, the incubation of one bacterial protein-enriched secreted fractions (PESF) with alternative species form same patient significantly increased biomass by 1.5–4.8 fold (*p* < 0.01), while similar trend was not observed among randomly cultured species. These data underscore the synergistic growth pattern between different bacterial species growing in the same niche and highlight the importance of further studies to aid the selection of antibiotics targeting polymicrobial biofilms.

**Supplementary Information:**

The online version contains supplementary material available at https://doi.org/10.1007/s00203-026-05020-3.

## Introduction

Polymicrobial infection is a hallmark of various chronic inflammatory conditions of the airways such as chronic rhinosinusitis (CRS), cystic fibrosis, and chronic obstructive pulmonary disease (COPD). In polymicrobial infections, interactions amongst various bacterial species can influence the severity of infection, the resulting inflammation and treatment success (Widder et al. 2024). *Staphylococcus aureus* and *Pseudomonas aeruginosa* can coexist in chronic infections via complex interactions and serve as drivers of infection, potentially impeding treatment success (García-Contreras et al. 2016). Unlike infections generated by a single species, the coexistence of *S. aureus* and *P. aeruginosa* in mixed infections is mostly related to enhanced pathogenic features and increased antibiotic resistance (Vestweber et al. 2024).

CRS is defined as a persistent inflammation of the nasal and sinus mucosa that compromises patients’ quality of life (DeConde and Soler 2016). Although the exact aetiopathogenesis of CRS is unclear, microbial infections are thought to be an important factor initiating and maintaining the inflammation (Vestweber et al. 2024). Recent studies have shown that polymicrobial biofilms coat the mucosal surface of the sinuses (Broderick et al. 2025). *S. aureus* and *P. aeruginosa* are amongst the most frequently isolated species in CRS patients and their co-infection is linked to a more severe disease with poorer treatment outcomes (XS et al. 2026). Their high prevalence in severe CRS patients implicates the potential of a coordinated action and enhanced virulence resulting in excessive inflammation, tissue damage, or barrier dysfunction (Kaliniak et al. 2024).

Biofilms are communities of bacteria encased in a self-produced polymeric matrix attached to surfaces. Bacterial cells embedded in biofilm matrix are protected from the host’s immune system and external factors such as antibiotics (Liu et al. 2024). Biofilms are therefore much more tolerant to antibiotics compared to their planktonic counterparts, in part due to the polymeric matrix which provides a diffusion barrier that prevents the antibiotics from penetrating deep within biofilms (Bin et al. 2025). Also, the metabolic activity of bacteria within the biofilm is reduced, further decreasing the effectiveness of antibiotics (Ahmad et al. 2025). Research findings indicate that communication between different bacterial species in polymicrobial biofilms plays a critical role in controlling the architectural organization of the biofilm as well as its metabolic properties (Trizna et al. 2020). In clinical infections, *S. aureus* and *P. aeruginosa* biofilms can co-exist. However, whether and how both species influence each other’s growth and biofilm formation is debated. *P. aeruginosa* produces a wide variety of quorum sensing molecules to coordinate the motility, expression of virulence factors and extracellular matrix formation (Scaffo et al. 2025). Many of those factors were shown to negatively affect *S. aureus* growth in vitro (Landa et al. 2024). Other studies however show an increase in biofilm complexity and pathogenicity when both species co-exist (Vestweber et al. 2024, Dehbashi et al. 2024). Hence, strain variation may influence interactions affecting growth patterns and virulence.

The polymicrobial nature of CRS is a major challenge to the clinician because of the difference in dominant species between patients and the need to target various species at the same time where both *S. aureus* and *P. aeruginosa* may have different susceptibilities to the antibiotics used in the treatment. Certain antibiotics may be useful in combating one pathogen, but are ineffective against the other, and their existence in biofilm form compounds this problem (Alammar et al. 2023).

Therefore, understanding the interactions of dominating species in the context of polymicrobial infections is crucial to optimize therapies that can effectively treat such infections in the sinonasal cavities. This study used *S. aureus* and *P. aeruginosa* isolated from the sinonasal cavities of CRS patients to evaluate the potential for reciprocal interaction between both species and their effect on biofilm formation and effectiveness of antibiotics (Tacconelli et al. 2018).

## Materials and methods

### Ethical approval and clinical isolate identification

#### Ethics approval

for the collection, storage, and use of clinical isolates from the sinonasal cavities of patients was granted by the Central Adelaide Local Health Network (CALHN) Human Research Ethics Committee (HREC) (CALHN Ref. 13604) and the Calvary Hospital HREC (Ref. 19-CHREC-E003), both in Adelaide, South Australia. This study was conducted in accordance with the principles of the Declaration of Helsinki. All patients had signed written informed consent. The diagnostic criteria for CRS were obtained from the European Position Statement on CRS (Fokkens et al. 2020). Matched *S. aureus* and *P. aeruginosa* were isolated from 3 distinct patients, and identity was confirmed by Matrix-Assisted Laser Desorption Ionization-Time of Flight Mass Spectrometry (MALDI-TOF MS, Bruker^®^ MBT, University of Adelaide, Adelaide, Australia) and stored at − 80 °C in tryptone soy broth (TSB, Oxoid, Basingstoke, UK) plus 20% (v/v) sterile glycerol until further use.

### Antimicrobial susceptibility and MIC determination

Antibiotics were selected to represent commonly used agents targeting Gram-positive and Gram-negative bacteria in experimental contexts. Ciprofloxacin was used for *P. aeruginosa*, amoxicillin for *S. aureus*, and amikacin for both species. The minimum inhibitory concentration (MIC) of antibiotics was determined using a broth microdilution method (Wiegand et al. 2008). *S. aureus* and *P. aeruginosa* clinical isolates grown overnight on 1.5% tryptic soy agar (TSA, Oxoid, Basingstoke, UK) plates were suspended in 0.9% normal saline at 0.5 MacFarland Units (1–2 × 10^8 CFU/mL) and diluted 1:100 in Mueller-Hinton Broth (MHB, Thermo Fisher Scientific, Waltham, MA, USA) in a 96-well plate containing serially diluted antibiotics with concentrations ranging from 128 µg/ml to 0.25 µg/ml; with positive (no antibiotics) and negative (media) controls. The microtitre plates were incubated at 37 C for 24 h. A 10 µL sample from the growth control was diluted and spread-plated onto 1.5% TSA plates in order to confirm the inoculum density before incubation. After incubation, the minimal concentration that inhibited visible growth of the microorganism was noted. Measurement of the optical density was conducted at 595 nm using a plate reader (BIO-RAD laboratories, CA, USA). All MIC assays were performed with 6 technical replicates and 3 independent culture replicates initiated from glycerol stocks of the same isolate. The MIC interpretation of susceptible and resistant breakpoints was based on the Clinical and Laboratory Standards Institute ((CLSI) 2023) and the European Committee on Antimicrobial Susceptibility Testing (EUCAST) (Testing 2019). Amikacin, amoxicillin and ciprofloxacin were from Sigma-Aldrich, St Louis, Mo, USA.

### *S. aureus* and *P. aeruginosa* biofilm formation

Overnight cultures of *S. aureus* and *P. aeruginosa* isolates grown on tryptic soy agar (TSA, Oxoid, Basingstoke, UK) plates were transferred into a sterile glass tube containing 0.9% sodium chloride and adjusted to a 1.0 ± 0.1 McFarland turbidity standard (approximately 3 × 10^8^ colony-forming units [CFU]/mL). The bacterial suspension was then diluted into nutrient broth at a 1:15 ratio. Subsequently, 180 µL of the final suspension was transferred to flat-bottom 96-well microtiter plates.

Single-species biofilms were established in a Transwell system (Corning, NY, USA) to model indirect interspecies interactions, with 13 mm coverslips placed in the basal chamber. A total of 100 µL of *S. aureus* or *P. aeruginosa* broth cultures were added to the upper chamber and 500 µL to the basal chamber across a 0.4 μm membrane. The 0.4 μm pore size allows diffusion of soluble factors but prevents bacterial crossover between chambers, enabling the assessment of indirect interactions only. This was experimentally verified by plating samples from the lower chamber onto selective agar plates for *S. aureus* (CHROMID *S. aureus* ELITE, bioMérieux) and *P. aeruginosa* (cetrimide agar), which showed no detectable growth. Plates were then incubated at 37 °C for 48 h on a rotating platform at 70 rpm (3D Gyratory Mixer) to allow biofilm formation.

In preliminary exploratory experiments, co-culture interactions were also assessed using laboratory reference strains (*S. aureus* ATCC25923 and *P. aeruginosa* PAO1), as well as representative clinical isolates (*S. aureus* C333 and *P. aeruginosa* C441). The corresponding data are provided in Supplementary Table S6 and Supplementary Figure S2.

### Preparation of protein-enriched secreted fractions (PESF)

The collection and quantification of bacterial biofilm PESF was carried out as described previously (Panchatcharam et al. 2020, Shaghayegh et al. 2023, Shaghayegh et al. 2023). Briefly, 48-hour biofilms were established in 6-well cell culture plates, followed by collection of supernatants and centrifugation at 1500 × g for 10 min at 4 °C. The supernatants were filtered through a 0.22-µm acrodisc^®^ syringe filter (Life Science, Fribourg, Switzerland) and concentrated using 3 KDa Pierce Protein Concentrators (Thermofisher, IL, USA). Concentrations were measured using the Nano-orange protein quantitation kit (Molecular Probes, Eugene, OR, USA) according to the manufacturer’s instructions. The fluorescence intensity was measured with excitation at 485 nm and emission at 590 nm using the FLUOstar Optima microplate reader (BMG Lab Tech). The preparation represents a cell-free, protein-enriched secreted fraction, which may also contain extracellular vesicles and membrane-associated components.

### Minimum biofilm eradication concentration (MBEC) assay

Established 48-hr biofilms were washed with phosphate-buffered saline (PBS). Amikacin was added in serial dilutions at concentrations from 2 to 64 µg/ml. Amikacin was selected for MBEC assays as all isolates demonstrated susceptibility under planktonic MIC conditions, enabling a standardized comparison of biofilm-associated antibiotic tolerance across strains. Sterile broth served as growth control. The plates were incubated at 37 °C for 24 h to determine the antibiotics’ minimum biofilm eradication concentration (MBEC) using crystal violet assays.

### Mixed-species and single species bacterial culture

#### MBEC assays for indirect interspecies biofilm interactions

Biofilms were established in a Transwell system (Corning), followed by aspiration of the media and adding 300 µL amikacin in media at a concentration of 16 µg/mL into both upper and lower chambers and incubation at 37 °C for a further 24 h. Media from lower chambers was serially diluted and plated onto 1.5% TSA plates followed by overnight incubation and enumeration of CFUs to evaluate contamination from the species from the upper chamber.

#### Crystal violet assay to determine biofilm biomass

Biofilm biomass was evaluated as previously described (Shaghayegh et al. 2023, Shaghayegh et al. 2024) with modifications. Biofilms were rinsed twice with PBS and coverslips from the lower chamber of transwells transferred into 24-well plates. Biofilms were stained with 180 µL or 500 µL of 0.1% (v/v) crystal violet solution per well (for 96-well plates and 24-well plates respectively) for 15 min at room temperature. The wells were rinsed thrice with sterile MilliQ water. Next, 180 µL/well (96-well plates) or 500 µL/well (24-well plates) of 30% acetic acid was added and incubated on a rotating platform (70 rpm) for 1 h. The absorbance of each well was measured spectrophotometrically at 595 nm using the FLUOstar OPTIMA microplate reader (BMG LABTECH). All experiments included a sterility control containing uninoculated nutrient broth. The experiments were repeated three times, with each repetition containing three technical replicates.

The percentage reduction in biomass value was calculated using the following formula:

$$ {\mathrm{Percentage}}~{\mathrm{Reduction}} = \left( {\frac{{{\mathrm{Initial~Biomass}}~ - ~{\text{Post - treatment~Biomass}}}}{{{\mathrm{Initial~Biomass}}}}} \right) \times 100\% $$

### Determination of PESF influence on biofilm biomass

The experimental groups included either the addition of PESF to planktonic cells during biofilm formation, or addition of PESF to established biofilms. In all experiments, control conditions were defined by the addition of sterile TSB to the upper chamber in place of PESF, which had been subjected to the same incubation and concentration procedures as the PESF preparations.

For PESF treatment during biofilm formation, concentrated PESF from single species bacterial biofilms were adjusted to 550 µg/mL and diluted 1:1 in 2x TSB. 90 µL of PESF was then mixed with 90 µL of a 1:15 dilution of 1MFU bacterial suspension in 96 well plates. These were then incubated at 37 °C for 48 h on a rotating platform at 70 rpm (3D Gyratory Mixer) to facilitate biofilm formation. For PESF treatment of established biofilms, 180 µL of a 1:15 diluted 1MFU bacterial suspension in 2x TSB was added to 96-well plates and incubated for 24 h at 37 °C on a rotating platform at 70 rpm (3D Gyratory Mixer) to allow biofilm formation. After 24 h, the media was aspirated, and 180 µL of the PESF dilution was added to give a final treatment concentration of 550 µg/mL. The plates were then incubated for an additional 24 h. Biomass measurement was conducted using the crystal violet assay.

### Statistical analysis

Statistical calculations were performed using Graph Pad Prism v 9.0.0 (121) (GraphPad Software, San Diego CA, USA). The Independent Samples t-test was used to assess the statistical differences between the means of two groups. For comparisons involving more than two groups, one-way ANOVA followed by Tukey’s multiple comparisons test was applied. The results were considered statistically significant when the p-value was < 0.05.

## Results

### *S. aureus* and *P. aeruginosa* reciprocally enhanced biofilm biomass when both species were isolated from the same patient

Three pairs of *S. aureus* and *P. aeruginosa* strains harvested from the sinonasal cavities of 3 CRS patients were used in experiments. Table 1 summarises the clinical and demographic characteristics of 3 adult patients with CRS. The patients varied in comorbidities, CRS phenotype, and disease severity scores. In all cases, *S. aureus* and *P. aeruginosa* were co-isolated from the same clinical sample, allowing for inter-patient comparison.

**Table 1:** Clinical and demographic characteristics of patients from whom CRS isolates were obtained

Patient ID	Age (years)	Sex	CRS Phenotype	Smoking History	Allergies	Asthma	GORD	DM	ADSS Score
6027	82	F	CRSsNP	Non-smoker	None	Yes	Yes	No	25
539	60	F	CRSwNP	Non-smoker	Yes	Yes	Yes	Yes	14.5
1415	53	M	CRSwNP	Unknown	None	No	Yes	Yes	Not recorded

*CRS*chronic rhinosinusitis; *CRSsNP*CRS without nasal polyps; *CRSwNP*CRS with nasal polyps; *GORD*gastroesophageal reflux disease; *DM*diabetes mellitus; *ADSS*Adelaide Disease Severity Score (Naidoo et al. 2013); *F*female; *M*male

**Fig. 1:**
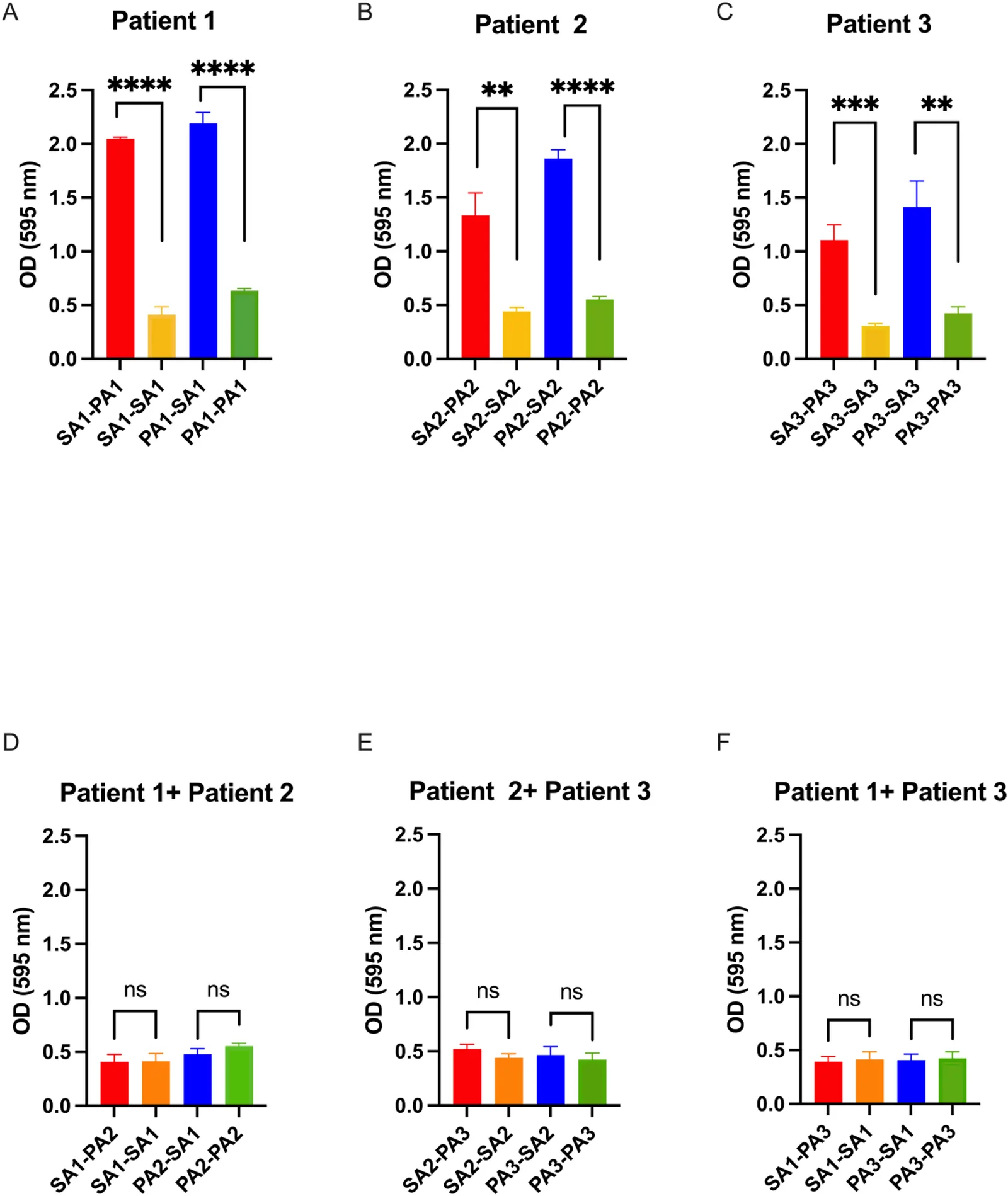
Biofilm biomass of *S. aureus* (SA) and *P. aeruginosa* (PA) in co-culture and monoculture conditions. (**A**) Biofilm biomass values of *S. aureus* and *P. aeruginosa* isolated from patients 1 (**A**), 2 (**B**), and 3 (**C**) in mixed cultures (SAx-PAx and PAx-SAx, where the biofilm biomass corresponds to that of *S. aureus* or *P. aeruginosa* respectively in each co-culture condition) and mono-culture (SAx-SAx and PAx-PAx in which the biofilm biomass of *S. aureus* or *P. aeruginosa* was measured in the lower chamber, with the same species present in the upper chamber). (**D**-**F**) Biofilm biomass of *S. aureus* and *P. aeruginosa* from different patients in co-culture and monoculture. **p < 0.01, ***p < 0.001, ****p < 0.0001, t-test; ns (not significant). SA: *S. aureus*, PA: *P. aeruginosa*, SA-PA: *S. aureus* in the lower chamber and *P. aeruginosa* in the upper chamber. PA-SA: *P. aeruginosa* in the lower chamber and *S. aureus* in the upper chamber

Bacterial biofilms of both species were alternatively co-cultured in the upper and lower chamber of Transwell plates for 48 h to determine the influence of each bacterial species on the biofilm thickness of the other species. Results showed that both *S. aureus* and *P. aeruginosa* biofilm biomass size increased by 3-5-fold when species were allowed to indirectly interact, compared to single species biofilms across the 3 pairs tested (*p* < 0.05) (Fig. 1A and C). Following these observations, we assessed whether biofilms under indirect interspecies interaction conditions formed by isolates from different patients would produce a similar effect. No significant increase in biomass was observed compared to single-species cultures (Fig. 1D–F). Detailed results for all pairwise combinations are provided in Supplementary Table S1.

### Determination of the minimum inhibitory concentration (MIC) of antibiotics against planktonic *S. aureus* and *P. aeruginosa* from CRS patients

To test whether the growth of both species could affect their antimicrobial susceptibility, we determined their individual susceptibility to antibiotics in planktonic forms before testing in biofilms. *S. aureus* and *P. aeruginosa* from the 3 patients were tested for their antimicrobial resistance to amoxicillin, ciprofloxacin and amikacin. Both species varied in their susceptibility to amoxicillin and ciprofloxacin, but all were susceptible to amikacin. MIC values against the 6 strains for amoxycillin, ciprofloxacin and amikacin are shown in Table 2. MIC breakpoints were interpreted according to the Clinical and Laboratory Standards Institute ((CLSI) 2023) and the European Committee on Antimicrobial Susceptibility Testing (EUCAST) (Testing 2019) and are provided in Supplementary Table S2.

**Table 2:** Minimum inhibitory concentration (MIC) and Susceptibility of bacterial strains to amoxicillin, ciprofloxacin, or amikacin

Antibiotics	Strains	MIC (µg/mL)	Antibiotic Susceptibility (S/I/*R*)
Amoxicillin	*S. aureus* (Patient 1)	8	I
	*S. aureus* (Patient 2)	2	S
	*S. aureus* (Patient 3)	16	R
Ciprofloxacin	*P. aeruginosa* (Patient 1)	16	R
	*P. aeruginosa* (Patient 2)	8	R
	*P. aeruginosa* (Patient 3)	1	I
Amikacin	*S. aureus* (Patient 1)	2	S
	*S. aureus* (Patient 2)	4	S
	*S. aureus* (Patient 3)	2	S
	*P. aeruginosa* (Patient 1)	1	S
	*P. aeruginosa* (Patient 2)	1	S
	*P. aeruginosa* (Patient 3)	4	S

*S* susceptible; *I*intermediate; *R*resistant. Interpretations are based on EUCAST and CLSI clinical breakpoints

### Minimum biofilm eradication concentration (MBEC) of amikacin targeting *S. aureus* and *P. aeruginosa* biofilm

**Fig. 2:**
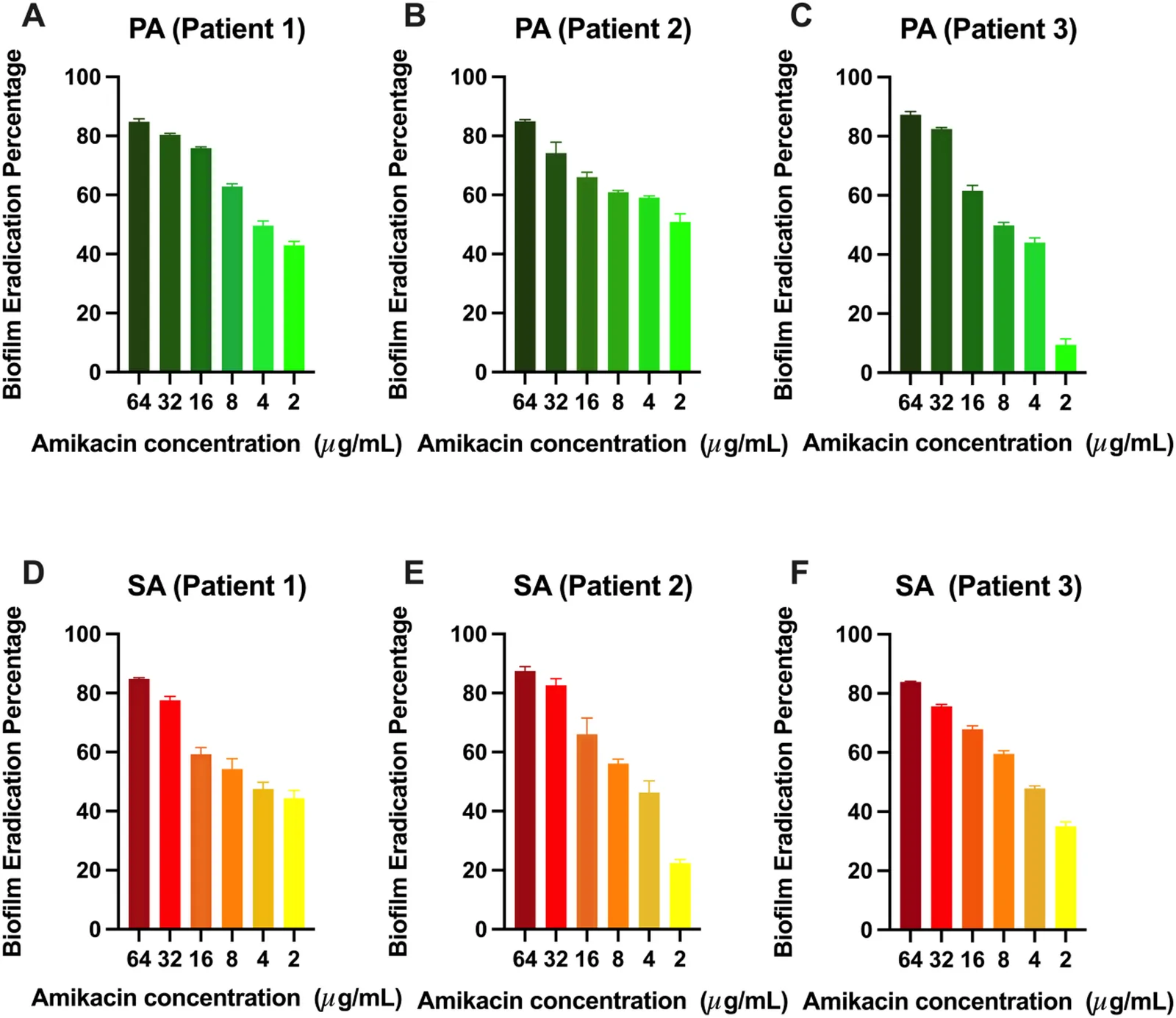
Minimum biofilm eradication concentration of amikacin for *S. aureus* and *P. aeruginosa* biofilms. Percentage of biofilm eradication under treatment with amikacin at concentrations of 2–64 µg/mL targeting *P. aeruginosa* and *S. aureus* biofilm from patient 1 (**A**, **D**), patient 2 (**B**, **E**) and patient 3 (**C**, **F**)

Given the sensitivity of both species to Amikacin in their planktonic mode of growth, we evaluated the susceptibility of their biofilms to amikacin in mixed and single species biofilms. As all isolates demonstrated susceptibility under planktonic conditions, amikacin provided a consistent baseline for assessing biofilm-associated tolerance across strains. The concentrations of amikacin ranged from 2 to 64 µg/mL and eradication was considered when the biofilm biomass was reduced by ≥ 80%. MBEC values were 64 µg/mL for all isolates except for *P. aeruginosa* from patient 3 and *S. aureus* from patient 2 where eradication was achieved with 32 µg/mL (Fig. 2A and F). 16 µg/mL of amikacin reduced the biofilm biomass in the range of 59.3%±2.2% − 67.9%±1.1% for *S. aureus* and by 61.5 ± 1.8% − 75.9%±0.4% for *P. aeruginosa* (Fig. 2A and F). The full set of MBEC values and biofilm biomass reduction percentages across all isolates are presented in Supplementary Table S3.

### *S. aureus* and *P. aeruginosa* biofilm were more tolerant to amikacin when grown in indirect contact with the alternate species

**Fig. 3:**
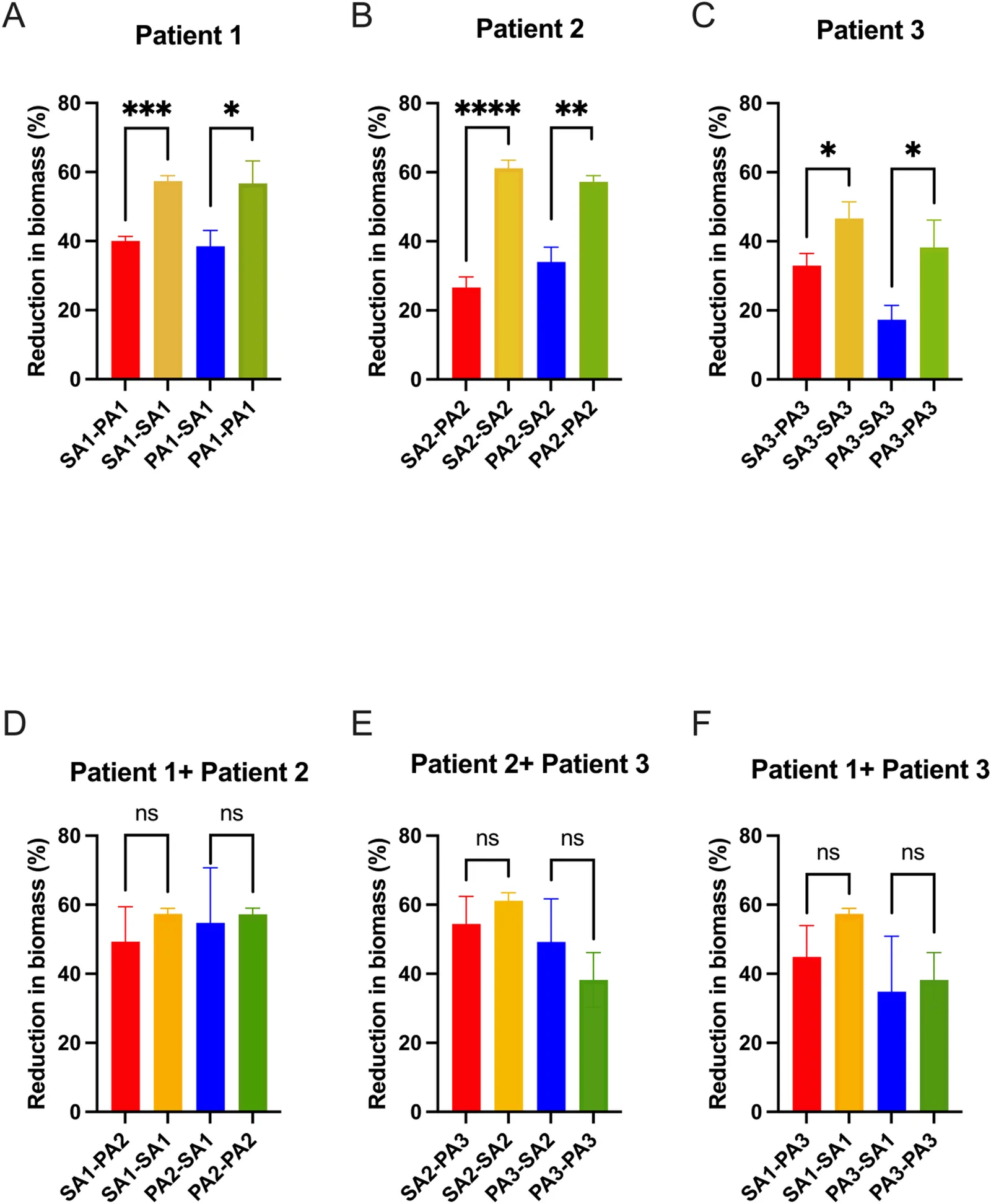
Percentage reduction in biofilm biomass following amikacin treatment. Biofilm biomass reduction (%) after 24 h exposure to 16 µg/mL amikacin was calculated for all conditions. In patient-matched conditions, co-cultures (SAx-PAx and PAx-SAx) showed lower percentage reduction compared to monocultures (SAx-SAx and PAx-PAx) across Patients 1–3 (**A**–**C**). Cross-patient comparisons are shown in (**D**–**F**), where this pattern was not observed. *p < 0.05, **p < 0.01, ***p < 0.001, ****p < 0.0001. t-test; ns (not significant). SA: *S. aureus*, PA: *P. aeruginosa*, SA–PA: *S. aureus* biomass (lower chamber) with *P. aeruginosa* in the upper chamber; PA–SA: *P. aeruginosa* biomass (lower chamber) with *S. aureus* in the upper chamber

Co-cultures of *S. aureus* and *P. aeruginosa* isolated from the same patient exhibited a significantly lower percentage reduction in biomass after 24-hour treatment with 16 µg/mL amikacin compared to single-species cultures for all 3 pairs, whereas individual species showed reductions in biomass of approximately 60%. This indicated an increased tolerance to antibiotics that had previously been active against each species’ biofilm under indirect interspecies contact (*p* < 0.05) (Fig. 3A-C). In contrast, this pattern was not observed in co-cultures of *S. aureus* and *P. aeruginosa* isolated from different patients, where there was no significant difference between single- and mixed-species cultures (Fig. 3D–F). Detailed quantitative results of biofilm biomass reduction and susceptibility to amikacin are summarized in Supplementary Table S4, with corresponding post-treatment OD values provided in Supplementary Figure S1.

### The impact of PESF from *S. aureus* and *P. aeruginosa* on biofilm biomass in CRS patients

**Fig. 4:**
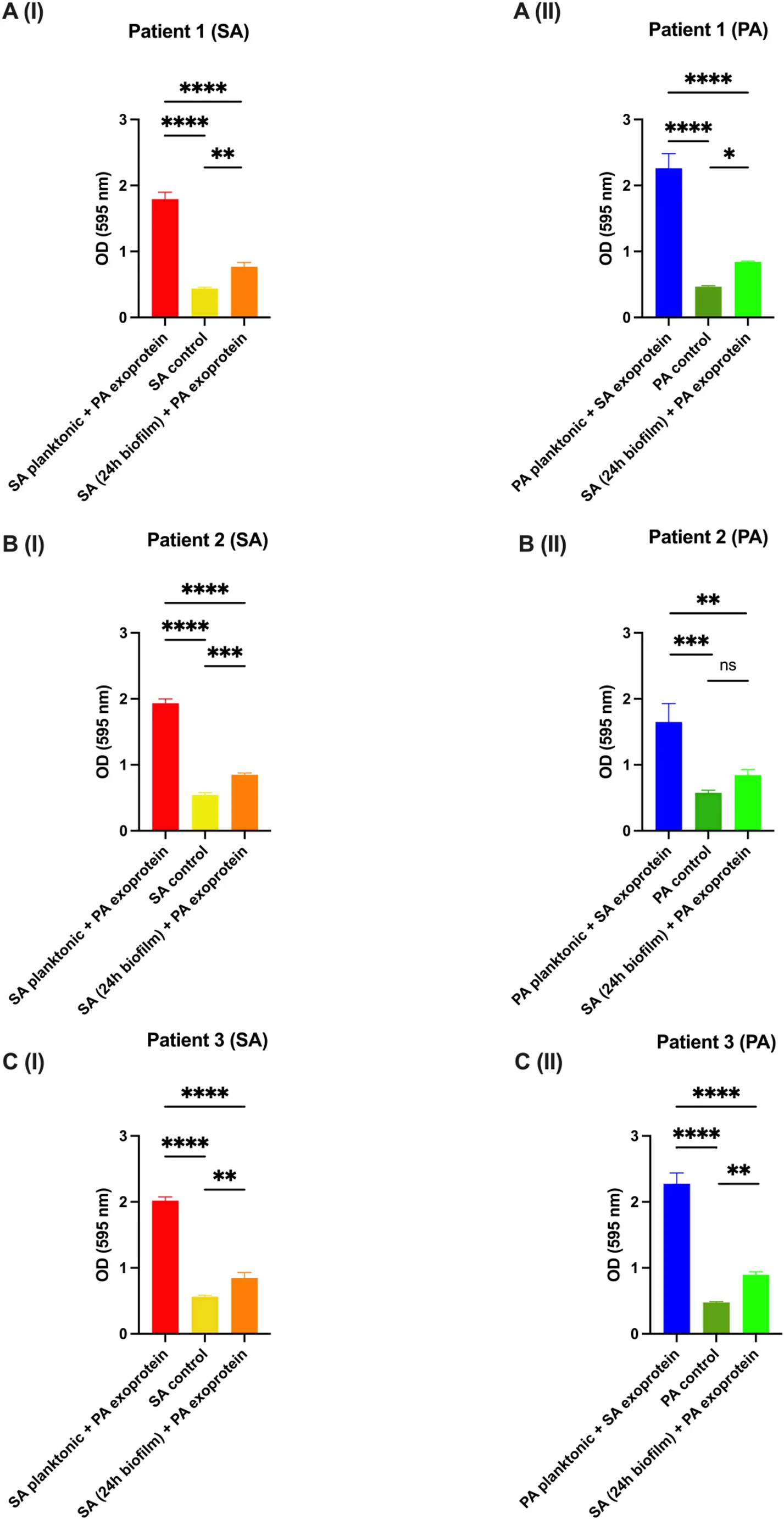
Effect of *S. aureus* and *P. aeruginosa* PESF on biofilm biomass. Biofilm biomass of *S. aureus* (**I**) and *P. aeruginosa* (**II**) isolates from patients 1–3 (**A**–**C**) following exposure to PESF from the alternate species and measured at OD (595 nm). Control groups consisted of *S. aureus* or *P. aeruginosa* cultured alone for 48 h. PESF were added either at the start of culture and maintained for 48 h, or added after 24 h of biofilm formation and co-incubated for a further 24 h. SA: *S. aureus*, PA: *P. aeruginosa*. Statistical significance was determined using one-way ANOVA followed by Tukey’s multiple comparisons test. Significance levels: *p < 0.05, **p < 0.01, ***p < 0.001, ****p < 0.0001, ns (not significant)

We then tested whether addition of biofilm PESF from either *S. aureus* or *P. aeruginosa* to the alternate species isolated from the same patient would affect the formation of biofilms. *S. aureus* or *P. aeruginosa* planktonic cells were incubated with concentrated PESF produced by *P. aeruginosa* or *S. aureus* from the same patient or broth control and grown to form biofilms for 48 h, followed by measuring their biomass. Results showed that the biofilm biomass of both species increased when incubated with PESF from the alternate species isolated from the same patient for all 3 patients (*p* < 0.05) (Fig. 4). In contrast, neither same-species PESF controls nor cross-patient PESF controls resulted in significant changes in biofilm biomass compared with monoculture controls (Supplementary Figure S3).

We next assessed whether PESF addition during early biofilm formation altered subsequent biomass accumulation. After biofilms were established for 24 h, PESF from the alternate species (same-patient isolate) were added for an additional 24 h. This 24 h add-on exposure showed a more limited effect than continuous exposure from the start of biofilm formation. In most conditions, biomass remained higher than monoculture controls without PESF, although this difference was not significant in all comparisons, while biomass remained lower than in biofilms grown in the continuous presence of PESF for 48 h (Fig. 4).

Comprehensive biomass data for all experimental conditions are provided in Supplementary Table S5.

## Discussion

This research investigated the phenotype of biofilms formed by *S. aureus* and *P. aeruginosa* from CRS patients. Our findings indicated that indirect interaction between *S. aureus* and *P. aeruginosa* isolated from the same patients resulted in an increased biofilm biomass and increased tolerance to antibiotics for both species when compared to monocultures. This may reflect reciprocal interactions that promote biofilm formation in both species within the host environment. Although competitive interactions can exist in planktonic phenotypes, chronic conditions such as persistent wounds may foster cooperative mechanisms between these bacteria (Briaud et al. 2020). This was supported by the augmented biomass noted both in *S. aureus* and *P. aeruginosa* when grown in co-culture, but only when those isolates were harvested from the same niche in the same patient. Indeed, compared to monocultures, no significant increase in biofilm biomass was observed when *S. aureus* and *P. aeruginosa* isolates from different patients were co-cultured. This indicated that specific interspecies interactions pivotal for biofilm augmentation, may have evolved through coexistence within the same host.

Previous in vitro studies have shown that *P. aeruginosa* exhibits an inherent advantage over *S. aureus* through the production of exoproducts that can inhibit *S. aureus* respiration and impair its growth (Roman-Rodriguez et al. 2025). In contrast, our study did not show a reduction of bacterial biomass when isolates from the same or from different patients were co-cultured. This could be due to the lack of direct contact between bacterial cells in this study, where we focused on indirect interactions between bacterial biofilms of both species.

Antibiotic resistance is a significant concern in the management of chronic infections, including CRS (Paramasivan et al. 2019). This study demonstrated substantial variability in antibiotic susceptibility among the isolates. This variability may complicate the interpretation of antimicrobial responses in polymicrobial settings. Our findings are consistent with previous reports demonstrating that interspecies interactions between *S. aureus* and *P. aeruginosa* can alter antimicrobial susceptibility and enhance biofilm-associated tolerance (Trizna et al. 2020, Beaudoin et al. 2017). In the present study, indirect interactions between patient-matched CRS isolates were associated with increased tolerance to amikacin compared with the corresponding monoculture biofilms. The increased tolerance may reflect biofilm matrix–mediated protection, which can limit antimicrobial penetration and contribute to bacterial persistence (Grooters et al. 2024), potentially in combination with interaction-dependent physiological adaptation. For instance, while higher matrix production may lead to increased stability and protection of bacterial cells within the biofilm (Fulaz et al. 2019), some bacteria develop metabolic cooperation that controls resources and resistance to exogenous effects (Zhang et al. 2024). Bacterial signaling, on the other hand, may play a significant role in making these biofilm structures more resistant by regulating gene expression in response to environmental changes and antibiotic pressure (Nourbakhsh et al. 2022). However, biofilms under indirect interspecies interaction conditions did not demonstrate altered tolerance to antibiotics when *S. aureus* and *P. aeruginosa* were derived from different CRS patients. These observations indicated an important role of the source of bacteria and host environment in biofilm formation and antibiotic tolerance (Derakhshan et al. 2021, Vaughn et al. 2020). These findings suggest enhanced antibiotic tolerance under patient-matched conditions. This may reflect coordinated interactions between *S. aureus* and *P. aeruginosa* associated with coexistence within the host environment (Beaudoin et al. 2017, Brandquist and Kielian 2025). Additionally, our data showed that biofilm PESF from the alternate species contribute to the increase in biofilm biomass of *S. aureus* or *P. aeruginosa*. These adaptive interactions may enhance bacterial survival and resistance to stress (Righi et al. 2023), and likely required prolonged coexistence within patient-specific environments. Longitudinal studies have demonstrated that *P. aeruginosa* can persist within the airway of a single patient for years, and in some cases even decades (Kuschnerow et al. 2023). Previous work by our team has shown that around 40% of *S. aureus* strains persist for > 6 months within the sinonasal cavities, associated with an increase in antibiotic tolerance over time (Houtak et al. 2023). Yet, further research is required to evaluate the molecular basis of reciprocal effects on biofilm biomass unique to *S. aureus* and *P. aeruginosa* harvested from the same niche.

The findings of this study demonstrated clinical relevance for the management of CRS and emphasised the necessity of targeted therapeutic approaches that consider specific microbial interactions and resistance mechanisms. Interactions between *S. aureus* and *P. aeruginosa* strains co-inhabiting the same niche may increase biofilm formation and antibiotic tolerance in vivo, thereby exacerbating the treatment-refractory nature of associated chronic infections. Therefore, treatment strategies should account for both the identity and antimicrobial resistance profiles of co-infecting species while also targeting biofilm disruption. Further research was warranted to determine whether disrupting inter-biofilm communication could reduce biomass and enhance the efficacy of existing antimicrobial therapies. Such approaches have been explored in other bacterial systems, where interference with quorum sensing has shown potential in reducing biofilm formation and improving antibiotic susceptibility (Alum et al. 2025).

This study had several limitations. The number of bacterial isolates was limited, and biological replication was based on independent culture preparations from the same isolate rather than multiple independently sampled isolates from each patient, restricting assessment of intra-patient variability. Only *S. aureus* and *P. aeruginosa* were examined, without consideration of host immunity or broader microbial diversity. The PESF preparation represented a protein-enriched secreted fraction that may have contained extracellular vesicles and other non-proteinaceous components; therefore, the observed effects could not be attributed exclusively to protein-mediated mechanisms. The molecular basis of the observed interaction-associated effects remains unclear. Future studies are warranted to define the molecular basis of these interactions using refined fractionation approaches, additional controls, and models incorporating host responses.

## Conclusions

The current study supports the notion of complex interactions between *S. aureus* and *P. aeruginosa* that are isolated from the same niche. The cohabitation of these bacteria within the same host environment might enhance PESF-mediated cooperative mechanisms associated with increased biofilm formation of both species and increased tolerance to antibiotics. Further studies are required to evaluate and identify the specific PESF that may mediate this interspecies coordinated enhancement of biofilm production.

## Supplementary Information

Below is the link to the electronic supplementary material.


Supplementary Material 1


## Data Availability

The data supporting the findings of this study are available from the corresponding author upon reasonable request. Due to institutional regulations, the raw data are stored on secure laboratory servers and cannot be deposited in a public repository. Data requests may be directed to the corresponding author, Prof. Sarah Vreugde, at sarah.vreugde@adelaide.edu.au, or to the co-author Xiaohan Sun at xiaohan.sun@adelaide.edu.au.
