# Supplementary material for: Reciprocal adaptation is critical in enhancing S. aureus and P. aeruginosa biofilm biomass
Source: Arch Microbiol. 2026 Jun 22;208(9):449. doi: 10.1007/s00203-026-05020-3 (PMC13287213; doi:10.1007/s00203-026-05020-3)
Supplement: Supplementary file 1 — Supplementary Material 1 [file 203_2026_5020_MOESM1_ESM.docx]

**Reciprocal adaptation is critical in enhancing *S. aureus* and *P. aeruginosa* biofilm biomass**

Xiaohan Sun ^a, b^, Clare M Cooksley ^a, b^, Muhammed Awad ^a, b^, Emma F Barry ^a, b^, Alkis J Psaltis ^a, b^, Peter-John Wormald ^a, b^, Sarah Vreugde ^a, b^

a Department of Surgery-Otolaryngology, Head and Neck Surgery, Central Adelaide Local Health Network, Woodville, South Australia, Australia

b Adelaide Medical School, Faculty of Health and Medical Sciences, The University of Adelaide, Adelaide, South Australia, Australia

Running title: Increased biomass of interacting *S. aureus* and *P. aeruginosa*

Corresponding author: Sarah Vreugde

Email: [sarah.vreugde@adelaide.edu.au](mailto:sarah.vreugde@adelaide.edu.au)

**Supplementary Information**


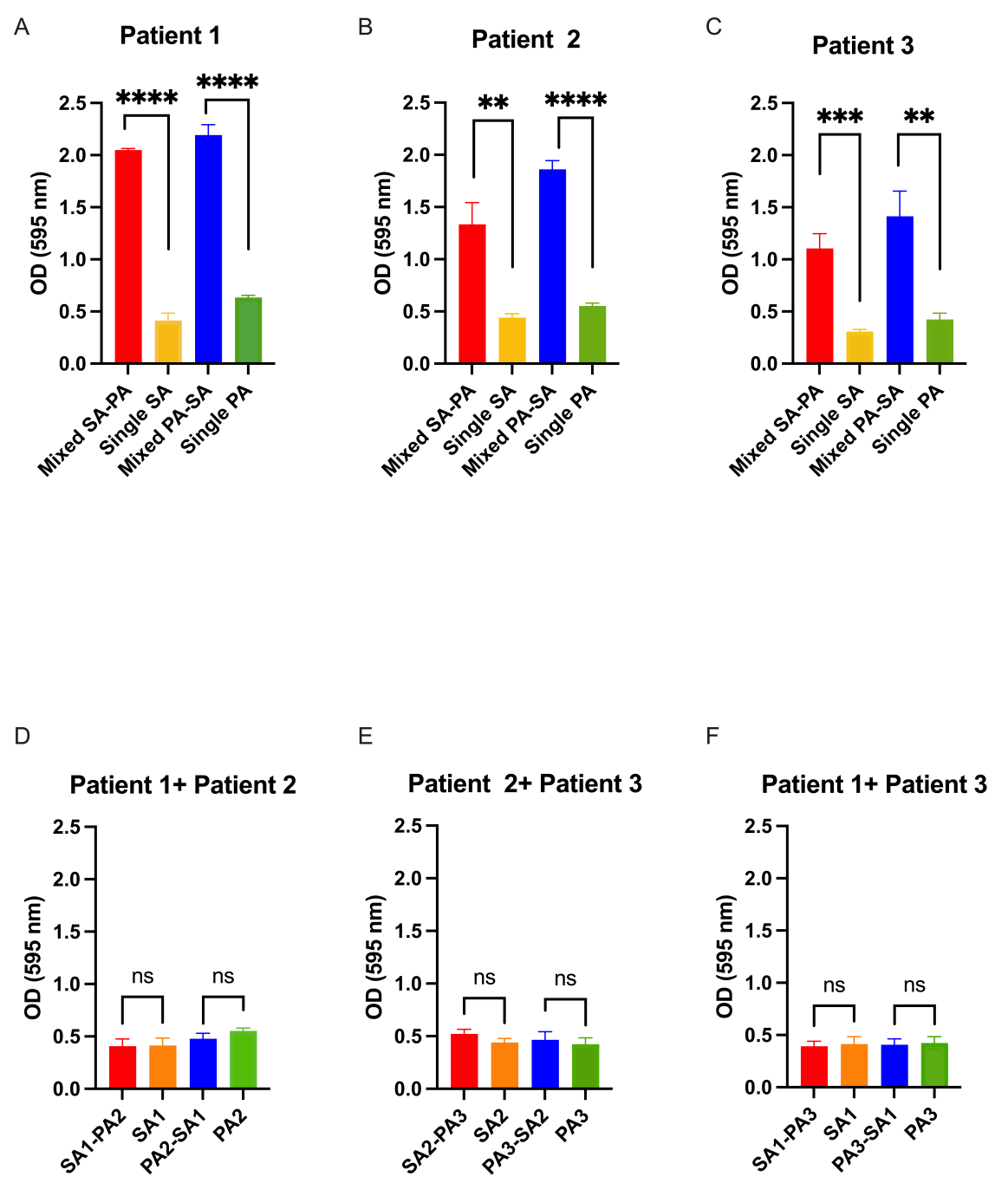


**Supplementary Figure S1.** Biofilm biomass of *S. aureus* (SA) and *P. aeruginosa* (PA) in co-culture and in single culture modes. (A) Biofilm biomass values of *S. aureus* and *P. aeruginosa* isolated from patients 1 (A), 2 (B), and 3 (C) in mixed cultures (mixed SA-PA and mixed PA-SA, where the biofilm biomass corresponds to that of *S. aureus* or *P. aeruginosa* respectively in each co-culture condition) and mono-culture (single SA and single PA, in which the biofilm biomass of *S. aureus* or *P. aeruginosa* was measured in the lower chamber, with the same species present in the upper chamber). (D-F) Biofilm biomass of *S. aureus* and *P. aeruginosa* from different patients in co-culture (mixed SA-PA and mixed PA-SA) and monoculture (single SA and single PA).

**p<0.01, ***p<0.001, ****p<0.0001, t-test; ns (not significant). SA: *S. aureus*, PA: *P. aeruginosa*, SA-PA: *S. aureus* is cultured in the lower chamber while *P. aeruginosa* is cultured in the upper chamber. PA-SA: *P. aeruginosa* is cultured in the lower chamber while *S. aureus* is cultured in the upper chamber.

**
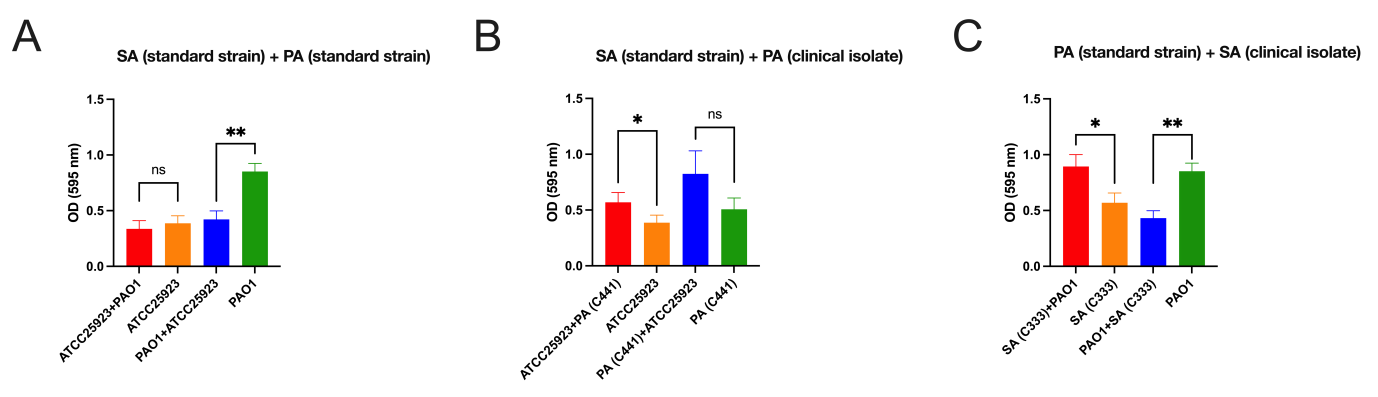
**

**Supplementary Figure S2.** Biofilm biomass under mono- and co-culture conditions. (A–C) Biofilm biomass of *S. aureus* (SA) and *P. aeruginosa* (PA) under mono-culture (single SA, single PA) and co-culture conditions (SA–PA and PA–SA). Panel A showed ATCC25923 (SA)–PAO1 (PA) strains, panel B showed SA standard (ATCC25923) with PA clinical isolate (C441), and panel C showed PA standard (PAO1) with SA clinical isolate (C333).

*p<0.05, **p<0.01, ns (not significant).





**Supplementary Figure S3.** Biofilm biomass following exposure to same-species and cross-patient protein-enriched secreted fractions (PESF) controls. (A–C) Biofilm biomass of *S. aureus* or *P. aeruginosa* after 48 h incubation with concentrated PESF derived from the same bacterial species isolated from the same CRS patient. (D–F) Biofilm biomass of *S. aureus* or *P. aeruginosa* after 48 h incubation with concentrated PESF derived from the alternate bacterial species isolated from a different CRS patient.

Statistical analysis was performed using t-test (A–C) and one-way ANOVA with Dunnett’s multiple comparisons test (D–F). ns, not significant (P > 0.05). SA: *S. aureus*, PA: *P. aeruginosa*, SA-PA: *S. aureus* is cultured in the lower chamber while *P. aeruginosa* is cultured in the upper chamber. PA-SA: *P. aeruginosa* is cultured in the lower chamber while *S. aureus* is cultured in the upper chamber.

***S. aureus* and *P. aeruginosa* Reciprocally Enhance Their Biofilm Biomass When Both Species Had Been Isolated from the Same Patient**

**Supplementary Table S1: Biofilm Biomass values as measured by crystal violet absorption at OD 595 nm**

| **Patient** | **Species in Lower Chambers** | **Species in Upper Chambers** | **Biofilm Biomass (OD ± SD)** |
| --- | --- | --- | --- |
| 1 | *S. aureus* | *P. aeruginosa* | 2.05 ± 0.01 |
| 1 | *S. aureus* | *S. aureus* | 0.41 ± 0.07 |
| 1 | *P. aeruginosa* | *S. aureus* | 2.39 ± 0.44 |
| 1 | *P. aeruginosa* | *P. aeruginosa* | 0.63 ± 0.02 |
| 2 | *S. aureus* | *P. aeruginosa* | 1.4 ± 0.13 |
| 2 | *P. aeruginosa* | *S. aureus* | 1.86 ± 0.08 |
| 2 | *S. aureus* | *S. aureus* | 0.44 ± 0.04 |
| 2 | *P. aeruginosa* | *P. aeruginosa* | 0.55 ± 0.03 |
| 3 | *S. aureus* | *P. aeruginosa* | 1.11 ± 0.14 |
| 3 | *P. aeruginosa* | *S. aureus* | 1.41 ± 0.24 |
| 3 | *S. aureus* | *S. aureus* | 0.31 ± 0.02 |
| 3 | *P. aeruginosa* | *P. aeruginosa* | 0.42 ± 0.06 |

**Determination of the Minimum Inhibitory Concentration (MIC) of Antibiotics Against *S. aureus* and *P. aeruginosa* from Chronic Rhinosinusitis Patients**

**Supplementary Table S2.** The breakpoints for susceptibility, intermediate susceptibility (semi-susceptibility), and resistance to amoxicillin, ciprofloxacin, and amikacin for *S. aureus* and *P. aeruginosa* established by the Clinical and Laboratory Standards Institute [1] and the European Committee on Antimicrobial Susceptibility Testing (EUCAST) [2].

| **MIC breakpoints** | **Amoxicillin**  ***S. aureus*** | **Ciprofloxacin**  ***P. aeruginosa*** | **Amikacin**  ***S. aureus P. aeruginosa*** | |
| --- | --- | --- | --- | --- |
| Susceptible (S)  Intermediate (I)  Resistant (R) | ≤2 µg/mL  4-8 µg/mL  ≥16 µg/mL | ≤0.5 µg/mL  1 µg/mL  ≥2 µg/mL | ≤16 µg/mL  32 µg/mL  ≥64 µg/mL | ≤16 µg/mL  32 µg/mL  ≥64 µg/mL |

**Minimum Biofilm Eradication Concentration (MBEC) of Amikacin against *S. aureus* and *P. aeruginosa* Biofilms**

**Supplementary Table S3: MBEC and Biofilm Reduction**

| **Patient** | **Species** | **MBEC (µg/mL)** | **% Reduction in biomass at 16 µg/mL** |
| --- | --- | --- | --- |
| 1 | *S. aureus* | 64 | 59.31 ± 2.20 |
| 1 | *P. aeruginosa* | 64 | 75.88 ± 0.38 |
| 2 | *S. aureus* | 32 | 66.02 ± 5.56 |
| 2 | *P. aeruginosa* | 64 | 66.01 ± 1.66 |
| 3 | *S. aureus* | 64 | 67.89 ± 1.14 |
| 3 | *P. aeruginosa* | 32 | 61.56 ± 1.80 |

***S. aureus* and *P. aeruginosa* biofilm are more tolerant to antibiotics when grown in indirect contact with the alternate species**

**Supplementary Table S4: Percent reduction in biofilm Biomass after exposure to Amikacin**

| **Patient** | **Lower Chambers** | **Upper Chambers** | **Biofilm Biomass after Amikacin Treatment (OD ± SD)** | **Reduction in Biofilm Biomass (%)** |
| --- | --- | --- | --- | --- |
| 1 | *S. aureus* | *P. aeruginosa* | 1.229 ± 0.028 | 40.02 ± 1.36 |
| 1 | *P. aeruginosa* | *S. aureus* | 1.472 ± 0.110 | 38.51 ± 4.58 |
| 1 | *S. aureus* | *S. aureus* | 0.219 ± 0.008 | 57.36 ± 1.61 |
| 1 | *P. aeruginosa* | *P. aeruginosa* | 0.274 ± 0.041 | 56.72 ± 6.50 |
| 2 | *S. aureus* | *P. aeruginosa* | 1.025 ± 0.043 | 26.62 ± 3.07 |
| 2 | *P. aeruginosa* | *S. aureus* | 1.228 ± 0.080 | 34.01 ± 4.30 |
| 2 | *S. aureus* | *S. aureus* | 0.171 ± 0.010 | 61.15 ± 2.32 |
| 2 | *P. aeruginosa* | *P. aeruginosa* | 0.237 ± 0.010 | 57.21 ± 1.85 |
| 3 | *S. aureus* | *P. aeruginosa* | 0.741 ± 0.039 | 32.96 ± 3.51 |
| 3 | *P. aeruginosa* | *S. aureus* | 1.168 ± 0.058 | 17.31 ± 4.09 |
| 3 | *S. aureus* | *S. aureus* | 0.180 ± 0.042 | 46.66 ± 4.79 |
| 3 | *P. aeruginosa* | *P. aeruginosa* | 0.261 ± 0.033 | 38.26 ± 7.90 |

**The Impact of Exoproteins from *S. aureus* and *P. aeruginosa* on Biofilm Biomass in CRS Patients**

**Supplementary Table S5a: Biofilm Biomass after 48 hours incubation with Exoproteins**

| **Patient** | **Species** | **Exoproteins Source** | **Biofilm Biomass after 48h (OD ± SD)** | **Adjusted p-value** |
| --- | --- | --- | --- | --- |
| 1 | *S. aureus* | *P. aeruginosa* (P1) | 1.795 ± 0.105 | <0.0001 |
| 1 | *P. aeruginosa* | *S. aureus* (P1) | 2.260 ± 0.223 | <0.0001 |
| 1 | *S. aureus* | None | 0.439 ± 0.018 | - |
| 1 | *P. aeruginosa* | None | 0.467 ± 0.015 | - |
| 2 | *S. aureus* | *P. aeruginosa* (P2) | 1.934 ± 0.065 | <0.0001 |
| 2 | *P. aeruginosa* | *S. aureus* (P2) | 1.648 ± 0.281 | 0.0006 |
| 2 | *S. aureus* | *None* | 0.542 ± 0.037 | - |
| 2 | *P. aeruginosa* | *None* | 0.577 ± 0.039 | - |
| 3 | *S. aureus* | *P. aeruginosa* (P3) | 2.019 ± 0.056 | <0.0001 |
| 3 | *P. aeruginosa* | *S. aureus* (P3) | 2.277 ± 0.163 | <0.0001 |
| 3 | *S. aureus* | *None* | 0.565 ± 0.022 | - |
| 3 | *P. aeruginosa* | *None* | 0.476 ± 0.013 | - |

## **Supplementary Table S5b: Biofilm Biomass After 24 hours incubation with Exoproteins**

| **Patient** | **Species** | **Exoproteins Source** | **Biofilm Biomass after 24h (OD ± SD)** | **Comparison with 48h adjusted p-value** | **Comparison with Mono-culture adjusted p-value** |
| --- | --- | --- | --- | --- | --- |
| 1 | *S. aureus* | *P. aeruginosa* (P1) | 0.768 ± 0.066 | <0.0001 | 0.0034 |
| 1 | *P. aeruginosa* | *S. aureus* (P1) | 0.842 ± 0.013 | <0.0001 | 0.0277 |
| 2 | *S. aureus* | *P. aeruginosa* (P2) | 0.849 ± 0.028 | <0.0001 | 0.0004 |
| 2 | *P. aeruginosa* | *S. aureus* (P2) | 0.844 ± 0.085 | 0.0029 | 0.2157 |
| 3 | *S. aureus* | *P. aeruginosa* (P3) | 0.847 ± 0.084 | <0.0001 | 0.0028 |
| 3 | *P. aeruginosa* | *S. aureus* (P3) | 0.896 ± 0.045 | <0.0001 | 0.0046 |

## **Supplementary Table S5c: Control Groups Biofilm Biomass after 48 hours incubation with Exoproteins from**

| **Patient** | **Species** | **Exoproteins Source** | **Biofilm Biomass after 48h (OD ± SD)** | **Comparison with Mono-culture** |
| --- | --- | --- | --- | --- |
| 1 | *S. aureus* | *S. aureus* (P1) | 0.399 ± 0.115 | ns |
| 1 | *S. aureus* | *P. aeruginosa* (P2) | 0.429 ± 0.133 | ns |
| 1 | *S. aureus* | *P. aeruginosa* (P3) | 0.497 ± 0.077 | ns |
| 1 | *S. aureus* | None | 0.458 ± 0.139 | - |
| 1 | *P. aeruginosa* | *P. aeruginosa* (P1) | 0.454 ± 0.039 | ns |
| 1 | *P. aeruginosa* | *S. aureus* (P2) | 0.405 ± 0.083 | ns |
| 1 | *P. aeruginosa* | *S. aureus* (P3) | 0.442 ± 0.035 | ns |
| 1 | *P. aeruginosa* | *None* | 0.460 ± 0.021 | - |
| 2 | *S. aureus* | *S. aureus* (P2) | 0.410 ± 0.098 | ns |
| 2 | *S. aureus* | *P. aeruginosa* (P1) | 0.467 ± 0.061 | ns |
| 2 | *S. aureus* | *P. aeruginosa* (P3) | 0.407 ± 0.072 | ns |
| 2 | *S. aureus* | None | 0.420 ± 0.099 | - |
| 2 | *P. aeruginosa* | *P. aeruginosa* (P2) | 0.494 ± 0.114 | ns |
| 2 | *P. aeruginosa* | *S. aureus* (P1) | 0.513 ± 0.149 | ns |
| 2 | *P. aeruginosa* | *S. aureus* (P3) | 0.528 ± 0.100 | ns |
| 2 | *P. aeruginosa* | *None* | 0.560 ± 0.115 | - |
| 3 | *S. aureus* | *S. aureus* (P3) | 0.444 ± 0.104 | ns |
| 3 | *S. aureus* | *P. aeruginosa* (P1) | 0.442 ± 0.078 | ns |
| 3 | *S. aureus* | *P. aeruginosa* (P2) | 0.452 ± 0.049 | ns |
| 3 | *S. aureus* | None | 0.452 ± 0.062 | - |
| 3 | *P. aeruginosa* | *P. aeruginosa* (P3) | 0.470 ± 0.094 | ns |
| 3 | *P. aeruginosa* | *S. aureus* (P1) | 0.488 ± 0.083 | ns |
| 3 | *P. aeruginosa* | *S. aureus* (P2) | 0.459 ± 0.106 | ns |
| 3 | *P. aeruginosa* | *None* | 0.501 ± 0.062 | - |

## **Supplementary Table S6. Biofilm biomass (OD595) under mono- and co-culture conditions using standard and clinical strains**

| **Strain combination** | **Species in lower chamber** | **Species in upper chamber** | **Biofilm biomass (OD595, Mean ± SD)** |
| --- | --- | --- | --- |
| ATCC25923 + PAO1 | *S. aureus* (ATCC25923, standard) | *P. aeruginosa* (PAO1, standard) | 0.34 ± 0.07 |
| ATCC25923 | *S. aureus* (ATCC25923, standard) | *S. aureus* (ATCC25923, standard) | 0.39 ± 0.07 |
| PAO1 + ATCC25923 | *P. aeruginosa* (PAO1, standard) | *S. aureus* (ATCC25923, standard) | 0.42 ± 0.08 |
| PAO1 | *P. aeruginosa* (PAO1, standard) | *P. aeruginosa* (PAO1, standard) | 0.85 ± 0.07 |
| ATCC25923 + PA (C441) | *S. aureus* (ATCC25923, standard) | *P. aeruginosa* (C441, clinical) | 0.57 ± 0.09 |
| ATCC25923 | *S. aureus* (ATCC25923, standard) | *S. aureus* (ATCC25923, standard) | 0.39 ± 0.07 |
| PA (C441) + ATCC25923 | *P. aeruginosa* (C441, clinical) | *S. aureus* (ATCC25923, standard) | 0.82 ± 0.21 |
| PA (C441) | *P. aeruginosa* (C441, clinical) | *P. aeruginosa* (C441, clinical) | 0.51 ± 0.10 |
| SA (C333) + PAO1 | *S. aureus* (C333, clinical) | *P. aeruginosa* (PAO1, standard) | 0.89 ± 0.11 |
| SA (C333) | *S. aureus* (C333, clinical) | *S. aureus* (C333, clinical) | 0.57 ± 0.09 |
| PAO1 + SA (C333) | *P. aeruginosa* (PAO1, standard) | *S. aureus* (C333, clinical) | 0.43 ± 0.07 |
| PAO1 | *P. aeruginosa* (PAO1, standard) | *P. aeruginosa* (PAO1, standard) | 0.85 ± 0.07 |

**All values represent mean ± standard deviation (SD) from three independent experiments.**
